# The olfactory neurobiology and chemical ecology of mosquito attraction to plant nutrient sources

**DOI:** 10.1098/rspb.2025.1806

**Published:** 2025-10-29

**Authors:** Melissa Leon Noreña, Sandeep Jandu, John Boyi, Lucia Ibarra Bouzada, Angela Rouyar, Iliano Coutinho Abreu, Fanying Chen, Omar S. Akbari, R. Jason Pitts, Jeffrey A. Riffell

**Affiliations:** ^1^ Department of Biology, University of Washington, Seattle, WA 98105, USA; ^2^ Department of Biology, Baylor University, Waco, TX 76798, USA; ^3^ School of Biological Sciences, Department of Cell and Developmental Biology, UC San Diego, La Jolla, CA 92037, USA

**Keywords:** mosquito, olfactory neurobiology, chemical ecology, odorant receptor, nectar, plant nutrients, mosquito–plant interactions, olfaction

## Abstract

Invasive mosquito species are key vectors of arboviral diseases, like dengue, zika and chikungunya, posing significant public health challenges worldwide. These issues are worsened by urbanization, climate change and insecticide resistance, driving research into new control methods. Adult mosquitoes are attracted to plant nutrient sources essential for flight and reproduction. However, few studies have examined the odours emitted by these sources, and little is known about the olfactory neurobiology of mosquito–plant interactions. This review synthesizes current knowledge on the broad classes of volatile compounds that modulate mosquito behaviour, focusing on the olfactory processes underlying mosquito responses to plant nutrient sources. We also discuss the application of neurogenetic tools for investigating the role of olfactory receptor genes and neural circuits in mosquito ecology. Finally, we explore how insights from these studies can inform and enhance mosquito control strategies, including developing synthetic lures for attractive toxic sugar baits and improved trapping and surveillance technologies. Defining the olfactory receptors, sensory neurons and neural circuits mediating attraction or repellency to plant odours is crucial for optimizing mosquito monitoring and control interventions.

## 1. Background: Significance of mosquitoes to global health

### (a) Mosquito vectors and emerging threats

Mosquitoes are among the most significant vectors of human diseases, transmitting pathogens that cause illnesses such as dengue, zika, chikungunya and malaria. Of these, malaria and dengue have historically recorded the highest case numbers and garnered significant global attention. Dengue, transmitted primarily by *Aedes aegypti* (L.), is endemic in over 100 countries and thought to infect 400 million annually [1,2], highlighting the need for more effective control strategies. *Aedes aegypti* is also responsible for the transmission of other significant arboviruses such as zika and chikungunya, and co-circulation of these diseases in some regions exacerbates morbidity and mortality rates [2]. Malaria, transmitted by *Anopheles* mosquitoes and caused by five *Plasmodium* parasite species, continues to be a devastating disease. In 2022 alone, it accounted for 249 million cases and 608 000 deaths—94% of which occurred in Africa [1,2].

Several interrelated factors drive the rising burden of mosquito-borne diseases. Climate change is regarded as the most significant threat to mosquito control [2]. Rising temperatures and shifting precipitation patterns influence mosquito development, geographical range and transmission dynamics. Additionally, rapid urbanization and population growth foster the spread of invasive mosquito species and create favourable conditions for disease outbreaks [3]. Invasive species such as *Aedes albopictus* (S.), *Ae. aegypti*, *Anopheles stephensi* and *Culex quinquefasciatus* are of particular concern due to their adaptability to urban environments and capacity to transmit multiple diseases [4]. *Anopheles stephensi’*s introduction to Djibouti in 2012 and expansion to other African countries have significant consequences for malaria control due to their role in transmitting malaria parasites in primarily urban locations [5]. Taken together, these converging factors highlight the urgent need for sustainable, integrated mosquito control strategies.

### (b) Population fitness

**Figure 1.**
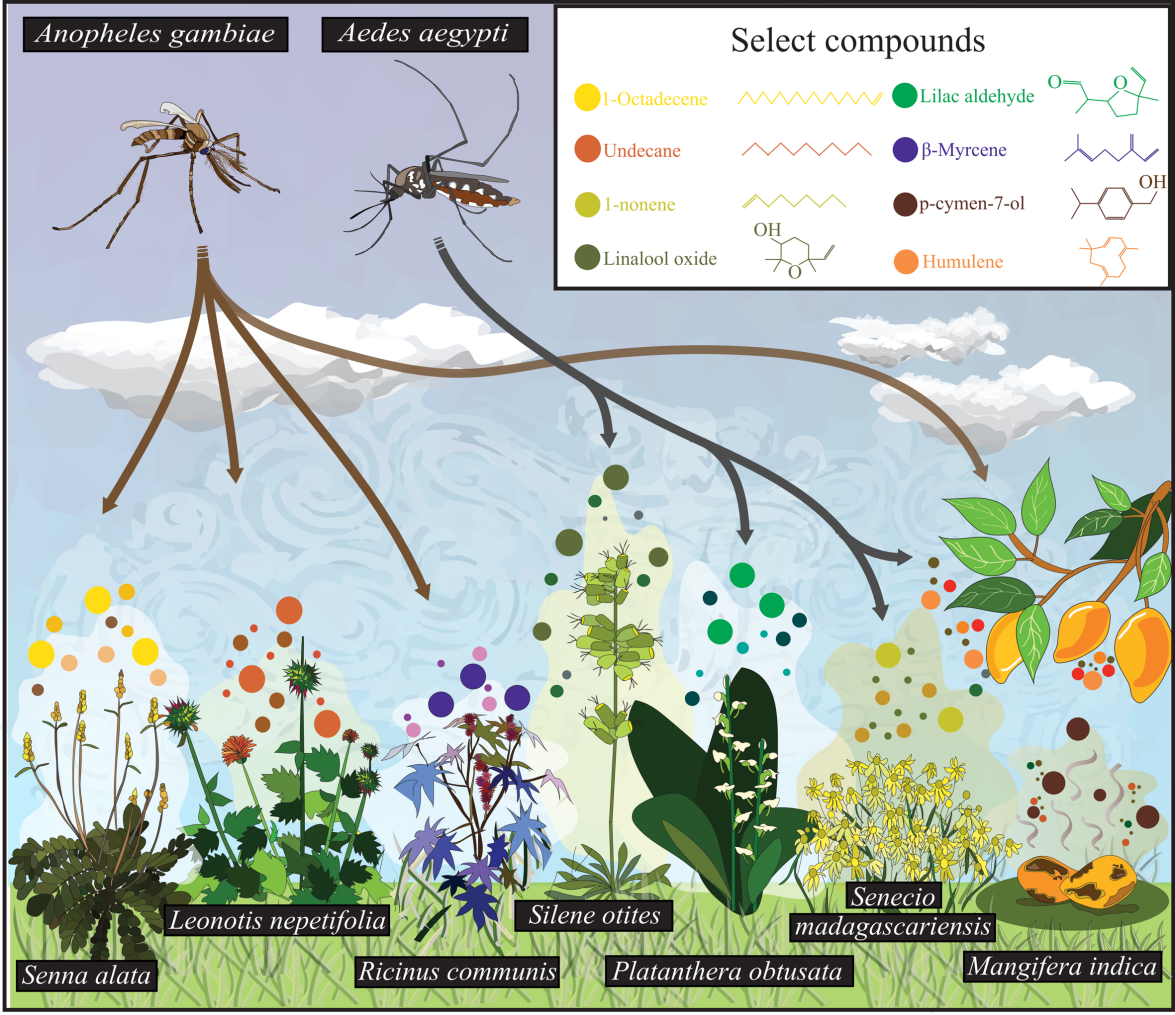
Olfactory behaviours by mosquitoes to plant-derived volatiles in their natural environment. *Aedes aegypti* and *Anopheles gambiae* are attracted to plant-emitted compounds. The highlighted plants (*S. alata, L. nepetifolia, R. communis, S. otites, P. obtusata, S. madagascariensis* and *M. indica*) emit distinct chemical profiles influencing mosquito behaviour. Identified odorants belong to diverse chemical classes, including hydrocarbons, terpenoids and phenolic derivatives. Upper left*: Ae. aegypti* (female) and *An. gambiae* (male) responding differentially to the floral odours. Upper right: colour-coded floral odorants emitted and their chemical structures. Bottom: selected plant species.

Nutrient-seeking behaviours mediated by plant odours significantly influence adult mosquito survivorship, producing direct and indirect consequences to disease transmission [6]. While blood feeding is widely recognized for its role in egg development, nutritional acquisition and pathogen transmission in females, not all mosquito species are blood feeding, and both sexes commonly feed on plant-derived nutrients [6]. These include sugars, carbohydrates, amino acids and phytochemicals, all of which can affect mosquito physiology in distinct ways (figure 1) [6]. Carbohydrates obtained from plants serve as the primary source of energy, supporting critical behaviours such as host seeking, oviposition, flight and reproduction [6]. For instance, in *An. stephensi*, glucose or trehalose was shown to elevate midgut pH, promoting *Plasmodium* gametogenesis and enhancing vector competence [7]. Similarly, plant-derived amino acids have been found to affect the reproductive fitness of *Ae. aegypti* [8].

Environmental factors such as nutrient availability and climate interact to further influence mosquito behaviour, physiology and population fitness, often in complex and context-dependent ways. On the one hand, a nutrient-poor environment can increase the biting rate and pathogen susceptibility of mosquitoes [9], whereas a nutrient-rich environment can enhance vectorial capacity by extending the longevity, abundance and overall reproductive fitness of mosquitoes [10]. In both situations, disease transmission may increase. For example, in more arid environments, *Ae. albopictus* that exhibited host-seeking behaviours were more likely to have sugar-fed than non-host-seeking mosquitoes [11]. Nutritional stress has been linked to an increase in susceptibility to dengue virus infections by suppressing immune signalling pathways [9]. In contrast, in plant nutrient-rich environments, the estimated vectorial capacity was 250-fold higher than in nutrient-poor environments for *Anopheles* mosquitoes [12,13].

## 2. Chemical ecology

Mosquito attraction to vertebrate hosts is comparatively well studied [14], whereas the necessary steps taken by mosquitoes to locate and feed from plant nutrient sources are less well characterized. Nonetheless, there is growing appreciation of the chemical ecology of plant nutrient sources [15–18]. Considering the diversity of chemical compounds mosquitoes process as they navigate to plants, characterizing odour emissions is essential to identifying both the potential common themes and distinctions across species and the requisite olfactory channels that mediate those responses [19].

### (a) Phytochemicals and physiological and behavioural effects

At close range, non-volatile phytochemicals, those ingested from plant tissues, are emerging as underexplored yet potentially influential modulators of mosquito physiology and behaviour. While most studies of plant extracts have focused on their toxicological properties across mosquito life stages [20], less attention has been paid to their potential beneficial effects on mosquito health and longevity. For instance, polyphenols, which are involved in diverse plant physiological functions, including plant defence and development, tended to increase *Ae. aegypti* lifespan by promoting autophagy of bacterial strains post-emergence via enhanced activity of AMP-activated protein kinase [21]. Additionally, a recent study demonstrated that *Ae. albopictus* can detect a diversity of these phytochemical compounds [22], suggesting they may be important for plant-feeding decisions more generally. With the ability to conduct calcium imaging from sensory neurons on the feeding appendages of *Ae. aegypti* mosquitoes [23], further characterization of different tastants is now possible.

### (b) Floral odour identification and behavioural correlates

At larger spatial scales, mosquitoes rely on plant-emitted volatile organic compounds (hereafter, odorants) to locate plant nutrient sources. Despite the diversity of potential plant nutrient sources, mosquitoes have been shown to exhibit preferences for certain flowers and fruits over others, mainly driven by the plants’ odour profile [24]. Most studies have consisted of laboratory or semi-field behavioural assays, correlating preferences with chemical analyses (typically gas chromatography with mass Spectrometry) of plant odorants. Comparatively few studies have identified bioactive volatiles from attractive plant nutrient sources using methods like gas chromatography with electroantennogram detection (GC-EAD).

In studies that correlated behavioural preferences with chemical analyses of attractive flower sources, flowers emit multiple classes of odorants, including monoterpenes, sesquiterpenes, alkanes, esters and aromatics. Nonetheless, a tentative pattern has emerged regarding the compounds that mediate mosquito attraction [19,25]. For instance, in *Culex pipiens pallens*, extracts from *Abelia chinensis R*. (Dipsacales: Caprifoliaceae) were attractive [26]. These extracts were rich in aromatic compounds, including benzaldehyde, benzyl alcohol, phenylacetaldehyde and phenylethyl alcohol, which have previously been identified as attractants for other mosquito species. Odorants emitted by attractive *Senecio madagascariensis* flowers for *Ae. aegypti* included a monoterpene, α-pinene and an alkene, 1-nonene [27]. Similarly, a landing bioassay study found that the aromatic compound, acetophenone, emitted from the flowering plant *Lobularia maritima*, attracted both male and female *Ae. aegypti*. For *An. gambiae*, *Parthenium hysterophorus* was reported as highly attractive, with key odorants identified as 1-octenol, β-pinene, *cis*-β-ocimene and α-caryophyllene [28]. In a much larger study examining the floral preferences of *An. gambiae*, attractive plants shared significant similarities in their odour profiles, including the monoterpenes β-pinene, (α- or β-) phellandrene and *cis*-β-ocimene, and the sesquiterpene α-caryophyllene [24]. Interestingly, in the latter study, an attractive floral outlier, *Senna occidentalis*, emitted a distinct profile dominated by carboxylic acids, perhaps analogous to the odour profile of humans [29] (figure 1).

Experiments using GC-EAD have provided both identification of the odorants detected by the mosquito’s antennae and insight into the strength of the olfactory responses to the isolated compounds [30]. *Anopheles gambiae* was electrophysiologically responsive to monoterpene compounds, including β-pinene, β-ocimene and D-limonene [31]. Building on these compound-specific findings, the antennal responses of three field-caught mosquito species in Kenya were examined [25]. Monoterpenes β-myrcene and (E)-β-ocimene were electrophysiologically active in all species and found in all tested host plants, while *Ae. aegypti* also responded to aromatics, *Aedes mcintoshi* to aldehydes and *An. gambiae* to sesquiterpenes and alkenes. Similar results were found in a survey of the GC-EAD responses of 11 different mosquito species in the Northwest US, including the non-native *Ae. aegypti* and *An. stephensi* [19]. A subset of odorants from the *Platanthera obtusata* flower, which is exclusively pollinated by mosquitoes, elicited responses, including the monoterpenes (β-myrcene, camphene, α- or β-pinene and lilac aldehyde) and aliphatic aldehydes (nonanal and decanal) [19].

While certain plant odorants may play a particularly large role in shaping mosquito attraction to sources of plant sugar, their presence alone is not sufficient to ensure attraction [19,32]. Rather, the concentration and proportions of these key odorants within the overall mixture are critical for attraction. Lahondère *et al.* [19] demonstrated that the floral species *P. obtusata*, which attracts *Aedes* spp. mosquitoes, emits a scent predominantly composed of the aliphatic aldehydes, with minimal amounts of the monoterpene lilac aldehyde. In contrast, the closely related *P. stricta*, pollinated by bees and moths, produces a similar fragrance but with a higher proportion of lilac aldehyde. This fragrance is repellent to mosquitoes. Similarly, work by Upshur *et al.* [33] analysed the odour composition of mosquito-associated ornamental plants and found that some of the most abundant compounds—such as germacrene D, caryophyllene and β-bisabolene—are also constituents of essential oils previously demonstrated to repel mosquitoes. These observations suggest that the role of such compounds in attraction versus repellency may depend on their proportions within the odour mixtures.

### (c) Fruit and seed-pod odours

In addition to floral chemical cues, mosquitoes are attracted to various fruit odours, including those from seedpods [34]. Mosquitoes feed from damaged fruits in natural environments [35], and many attractive toxic sugar baits (ATSBs) are formulated using fruit-based attractants [34]. Compared with floral volatiles, less is known about the specific fruit odorants mediating mosquito behaviour. Studies on mosquito fruit attraction have focused on the context of improving integrated vector management by assessing the behavioural valence of fruit odours as attractant lures [36]. These studies have often used processed materials (e.g. fruit concentrates or juices) that do not reflect the natural odour emissions from fruits. Nonetheless, results of these studies have provided insight into the fruits that are attractive to mosquitoes, with the caveat that future studies are necessary to identify the bioactive and attractive fruit odorants. For example, terpenes from mango juice attracted *An. gambiae*, with key compounds humulene, (E)-caryophyllene and terpinolene eliciting strong antennal and behavioural responses [37].

### (d) Host and plant nutrient odours

The importance of carboxylic acids, ketones and alcohols in the odours from blood hosts has been a long-time focus for the development of traps and identifying individuals who are super attractors to anthropophilic mosquitoes, including *Ae. aegypti* and *An. gambiae* [14]. Nonetheless, many compounds are shared between plant nutrient sources and vertebrate blood hosts. For example, *Anopheles* and *Aedes* mosquitoes are attracted to human hosts and plants (e.g. *Lantana camara* and *P. obtusata*) that share compounds in their odours, such as 1-octen-3-ol and aliphatic aldehydes [14,15]. Carbon dioxide is also emitted from plants during respiration, which synergizes with the plant odorants [38]. Although plant nutrient sources are often distinct in their odour profile by lacking carboxylic acids, the significant overlap raises the question of how the mosquito olfactory system processes the odours.

## 3. Sensory basis for plant odour detection and processing

The detection and processing of plant odours scale from the receptor to the neuronal circuits that process the odour information, and ultimately, mediates behaviour. Diverse families of chemoreceptors facilitate the transduction of chemical information from sensory neurons in peripheral organs to the central brain. These receptors, including odorant receptors (ORs), ionotropic receptors (IRs) and gustatory receptors (GRs), each play important roles in detecting distinct classes of compounds. For example, IRs are involved in detecting carboxylic acids, temperature and humidity, whereas GRs are involved in detecting sugars, alkaloids and CO_2_. In contrast, ORs are primarily receptors responsible for detecting plant volatiles and are likely to be especially important for plant nutrient foraging behaviours [39]. The behavioural attraction of mosquitoes to plant odorants, including those from fruits and flowers, also depends on the sensory architecture and neural coding strategies that govern odour detection and discrimination. Previous articles have reviewed odorant reception and the peripheral olfactory system of mosquitoes [40–42]. Here, we focus on the odorant receptors, neuroanatomy and how the peripheral and central olfactory areas represent and encode olfactory information, with a special emphasis on plant odorants.

### (a) Structural and evolutionary patterns of odorant receptor–ligand interactions

**Figure 2.**
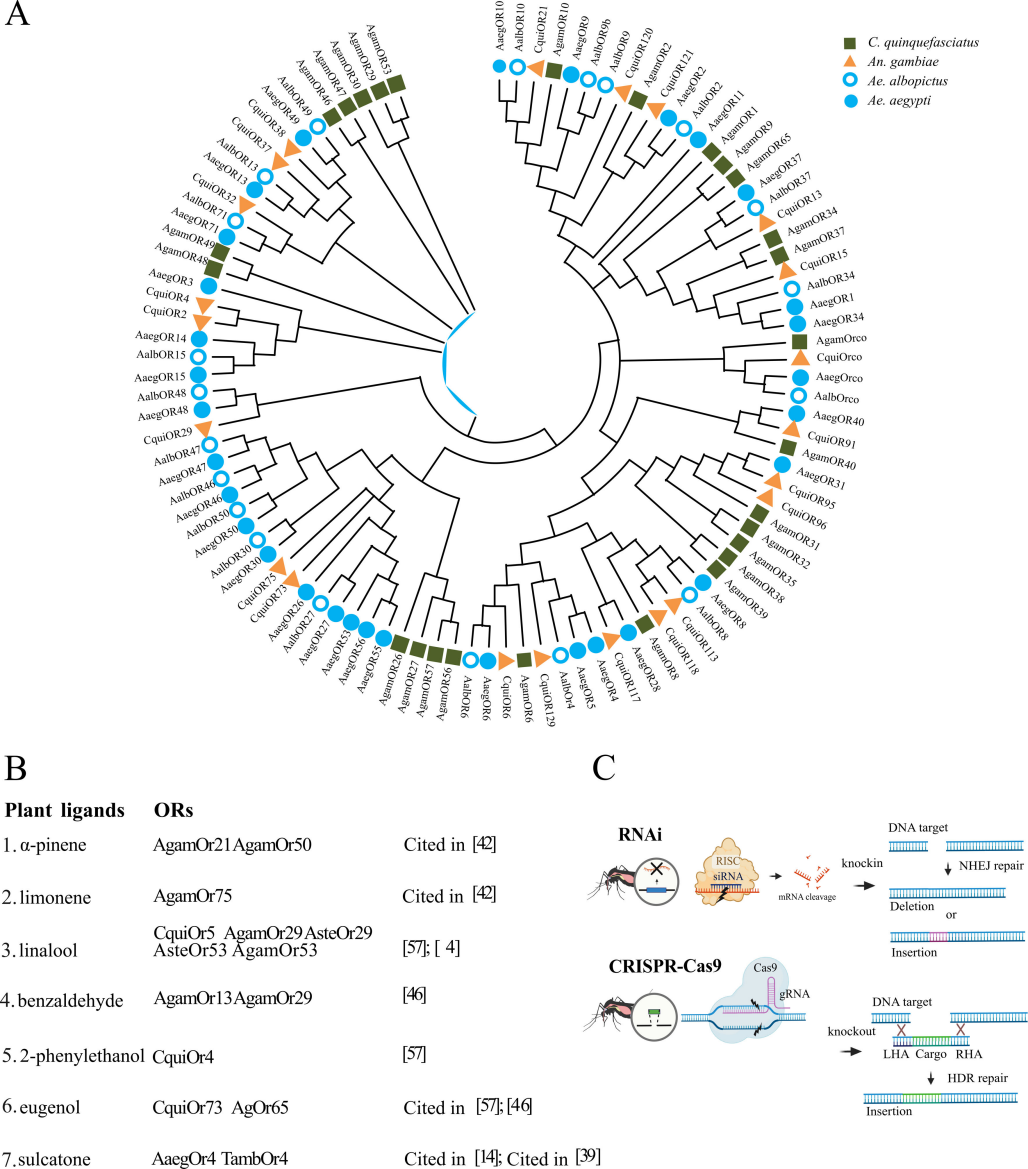
Evolutionary relationships across conserved mosquito odorant receptors and genetic approaches to study odorant receptor functions. (A) Maximum likelihood bootstrap consensus tree (500 replicates) showing the evolutionary history of the most conserved ORs in four mosquito species: *An. gambiae* (Agam, green square), *Cx. quinquefasciatus* (Cqui, orange triangle), *Ae. aegypti* (Aaeg, filled blue circle) and *Ae. albopictus* (Aalb, open blue circle). Branches with less than 50% confidence were collapsed. OR amino acid sequences were downloaded from Vectorbase, and the tree was generated using MEGA12. (B) List of plant ligands and ORs sensitive to those compounds. (C, top) RNA interference (RNAi) reduces gene expression. Small interfering RNA (siRNA) guides the RNA-induced silencing complex (RISC) to the target mRNA, leading to its cleavage. (C, bottom) Clustered Regularly Interspaced Short Palindromic Repeats (CRISPR)/Cas9-mediating gene disruption. Gene knockout is the result of deletion, insertion or single-nucleotide polymorphisms mediated by the non-homologous end-joining repair mechanism (NHEJ). Gene knock-in by DNA cassette insertion is mediated by homology-directed repair (HDR). This approach utilizes a donor template containing homology arms (Left Homology Arm, (LHA )and Right Homology Arm, (RHA)) to facilitate precise DNA insertion. Panel C modified from [50].

Structurally, ORs are transmembrane proteins that form tetrameric complexes with a single tuning OR subunit forming a ligand-gated ion channel with three subunits of the highly conserved co-receptor, Orco [43]. The strong evolutionary conservation of Orco is demonstrated by the functional redundancy between *An. gambiae* and *Drosophila melanogaster* despite their divergence over 250 Ma [44]. In contrast, the OR gene family is among the most divergent gene families in insects, and by extension, mosquitoes [45]. Phylogenetic analyses have revealed patterns of OR clustering and divergence within mosquito species [46–48]. Very few conserved ORs are shared across mosquito species, potentially reflecting constraints that preserve receptors crucial for olfactory functions over long evolutionary time scales [49]. These conserved ORs are contrasted by major species-specific expansions, which may allow adaptation to unique ecological requirements (figure 2A,B). Single gene orthologs shared between Anophelines and Culicines are rare, with the indolergic receptors OR2 and OR10, and the 1-octen-3-ol receptors (e.g. OR8), providing the clearest examples of functional conservation across subfamilies (figure 2A, electronic supplementary material, Table S1) [51,52].

Although an ever-increasing number of mosquito genomes are publicly available, surprisingly few studies have attempted broad ligand–receptor functional characterizations. Overall, ORs exhibit a wide range of activation, with some narrowly tuned to specific volatiles and others responding to a broader array of ligands. This flexibility may enable mosquitoes to adapt their foraging strategies to a wide range of floral environments, suggesting that ORs are adapted to balance between precision and versatility [39]. The most comprehensive investigations to date have examined the OR family in *An. gambiae* [45,53]. Although not specifically focused on plant volatiles, the diverse chemical activation of *An. gambiae* ORs revealed in these studies include several ORs that are tuned to potential plant attractants. For example, AgamOR35 was highly selective to fenchone, a common volatile in plant essential oils [54], while many other AgamORs responded to alcohols, ketones and indoles [45,53]. A recent study characterized the function of multiple ORs in *Ae. aegypti*, many of which were activated by plant volatiles, although their roles in plant nutrient seeking remain unknown (electronic supplementary material, Table S1) [39].

Despite these limitations in our knowledge of OR function, some interesting ligand–receptor relationships hint at mosquito plant odorant sensitivities that in some cases may also facilitate behavioural responses to other natural resources. For example, OR29 and OR53, receptors conserved across Anopheline genomes, are activated by the plant volatile, linalool, but with distinct thresholds for linalool stereoisomers [55]. Another important example of enantiomeric sensitivity is AaegOR8, which is expressed predominantly in the maxillary palps of both sexes and is highly specific to the R(-) isomer of 1-octen-3-ol, a well-characterized volatile associated with floral, fungal and human emissions [39,53,56]. The broad conservation of 1-octen-3-ol receptors in both hematophagous and non-hematophagous species suggests multiple roles for this receptor in behavioural responses [56]. In *An. gambiae*, AgamOR8 is responsive to 1-octen-3-ol, AgamOr65 to 2-ethylphenol and AgamOr2 to indole, all of which occur in both floral and host-associated odour profiles [46,53], further blurring the lines between modes of resource acquisition. Ultimately, more genetic, neurophysiological and behavioural studies are needed to elucidate the importance of specific ORs on mosquito chemical ecology and to inform potential new surveillance and control strategies.

### (b) Odorant receptor function and behavioural outcomes

Genetic manipulation of odorant receptors in mosquitoes has become a powerful approach for elucidating the roles of plant volatiles in attraction, repellency and sensory processing [50]. Two primary techniques—RNA interference (RNAi) and CRISPR-Cas9 genome editing—are commonly employed to dissect olfactory function. RNAi knockdown involves introducing double-stranded RNA (dsRNA) to trigger the degradation of specific messenger RNA (mRNA) transcripts and results in transient gene silencing (figure 2C). This method is useful for assessing short-term effects on olfactory function and behaviour without altering the genome. CRISPR-Cas9-mediated knockout enables permanent disruption of target genes by introducing double-strand breaks at specific genomic loci. These breaks are typically repaired by error-prone non-homologous end joining (NHEJ), resulting in small insertions or deletions (indels) that introduce frameshift mutations and disrupt gene function (figure 2C). Together, advanced genetic tools such as knockdown, knockout and knock-in strategies enable precise manipulation of olfactory genes and neuronal pathways, allowing for a direct link of olfactory receptors to mosquito behaviour.

Clear examples of how RNAi knockdown of ORs can be functionally linked to behavioural responses to specific plant volatiles come from studies in *Cx. quinquefasciatus*. CquiOR4 and CquiOR5 have been linked to plant volatile repellency [57]. CquiOR4 is enriched in the female antennae, while CquiOR5, although belonging to the same phylogenetic cluster as CquiOR4, is not specific to the antennae [57]. RNAi-mediated knockdown of either CquiOR4 or CquiOR5 reduced the repellency of *Cx. quinquefasciatus* to naturally occurring plant volatiles, although the effect of CquiOR5 knockdown was not statistically significant [58]. More specifically, CquiOR4 mediates repellency to 2-phenylethanol, while CquiOR5 is involved in responses to linalool and para-menthane-3,8-diol [58].

CRISPR/Cas9-mediated knockout has also been shown to be an effective strategy for targeting ORs that transduce plant- and microbe-related compounds. For example, the highly conserved receptor, OR49, is expressed in the cpC neuron of capitate peg sensilla in the maxillary palps of Culicinae mosquitoes but not in Anopheline [59]. The latter subfamily of mosquitoes expresses OR28 in the cpC neuron [60]. OR49 is activated by plant volatiles associated with mosquito repellency, such as camphor, borneol and other monoterpenes [59]. Electrophysiological investigations showed strong cpC neuronal activity to borneol in *Ae. aegypti*, *Ae. albopictus* and *Cx. pipiens* but not in *An. gambiae*. In *Ae. aegypti,* Or49 gene knockout line was also devoid of cpC activity upon borneol exposure [59]. Behavioural investigation also demonstrated that the mosquito *Ae. aegypti* is repelled by borneol [59]. All together, these functional and behaviour studies demonstrated that mosquitoes that diverged over 40 Ma display conserved olfactory pathways that evoked repellency behaviour upon exposure to the plant-based repellents [59].

CRISPR/Cas9-mediated knock-in approaches expand the toolkit for precise genetic modifications, enabling researchers to study the chemosensory basis of plant volatile detection in vector mosquitoes with unprecedented detail (reviewed in [50]). For instance, CRISPR-mediated insertion of calcium sensors, such as genetically encoded calcium indicator (GCaMP), or neurotransmitter sensors, like calcium modulated photoactivatable ratiometric integrator (CaMPARI), would allow for the imaging of neuronal activity in response to plant volatiles, providing direct evidence of odour-evoked responses in olfactory sensory neurons (OSNs) [19,61]. Additionally, integrating reporter genes and fluorescent markers facilitates the visualization and mapping of sensory circuits involved in detecting nectar and plant volatiles [62]. When combined with binary expression systems, such as the binary gene expression system QF/QUAS system, knock-in strategies become even more powerful. These systems involve knocking in a transcriptional activator QF into specific neuronal genes, enabling it to bind its respective promoter QUAS and drive targeted gene expression in defined cell types [63,64]. This approach enables precise, cell-type-specific manipulation of olfactory signalling pathways, providing invaluable insights into how mosquitoes detect and respond to plant nutrient volatiles.

#### (i) Peripheral representation and processing of plant odours

**Figure 3.**
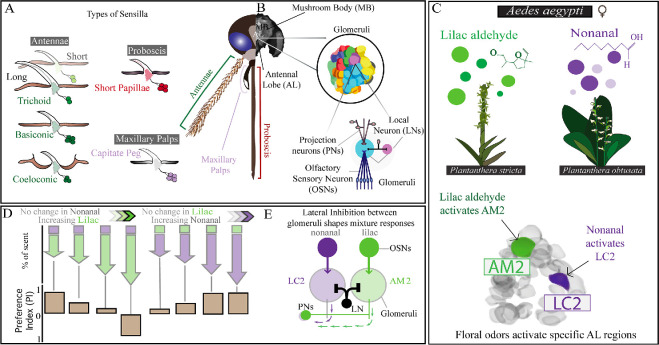
Behavioural and neural responses to plant-derived volatiles in mosquitoes. (A) Olfactory sensilla types involved in processing plant volatiles in mosquitoes (e.g. *Ae. aegypti*). (B) The organization of the mosquito olfactory system, including the olfactory periphery (antennae, maxillary palps and proboscis), and loci in the central nervous system, including the antennal lobe (AL) with associated glomeruli and neurons, and higher brain areas like the mushroom bodies. (C) Nonanal and lilac aldehyde emitted from *P. obtusata* and *Plantathera stricta* flowers, respectively, activate distinct glomeruli in the *Ae. aegypti* AL. (D) Mosquito preference when exposed to mixtures of lilac aldehyde and nonanal. (E) Lateral inhibition between glomeruli modulates responses to odours. Panels C and D were modified from Lahondère *et al.* [19].

The mosquito’s peripheral olfactory system is primarily distributed across three sensory appendages: the antennae, the maxillary palps and the proboscis (figure 3A) [65]. Specific cuticular structures called sensilla, located on their surfaces, house OSNs responsible for detecting volatiles. A single sensillum can host between one and four OSNs [66]. Volatiles enter the sensilla through pores or slits and are bound by odorant-binding proteins through the sensillar lymph to reach and bind to the ORs. Upon activation of these receptors, a series of molecular cascades converts the chemical signal into an electrical signal in the form of a spike, which is transmitted to the antennal lobes (ALs).

On the olfactory periphery, there are different morphological subtypes of sensilla, including trichoid, basiconic and coeloconic sensilla (figure 3A). Across mosquito species, there have been increasing studies on the functional characterization of the odorant sensory neuron tuning [60,67,68]. However, work is needed to determine whether sensillar morphology and function are conserved across species, particularly for the detection of plant odours. Nonetheless, functional studies of the capitate peg (basiconic) sensilla on the maxillary palp of *Ae. aegypti* [69], *Cx. quinquefasciatus* [68] and *An. gambiae* [60] show these sensilla are highly sensitive to 1-octen-3-ol and to the terpene borneol [59]. Trichoid sensilla are the dominant sensilla on the antenna of *An. gambiae*, *Ae. aegypti* and *Cx. quinquefasciatus* [70], and the trichoid subtypes, such as the blunt-tipped and sharp-tipped, can show diverse responses to odorants, including those emitted from plants. Examples include a long trichoid sensilla subtype on the *Ae. aegypti* antenna, which selectively responds to a high concentration of β-myrcene [71]. In *Ae. aegypti* and *Cx. quinquefasciatus*, short trichoid subtypes show strong responses to diverse odorants, including terpenes, esters, aldehydes and carboxylic acids. For *An. gambiae*, short trichoid sensilla also show broad response tuning, with particularly strong responses to carboxylic acids, aromatics and alcohols. Short sensilla on the proboscis in *Anopheles* mosquitoes respond to aldehydes, terpene, acetophenone-like compounds and carboxylic acid [72,73].

Despite the closing gaps in our understanding of OSN and associated sensilla, research on chemosensory receptor expression may shed additional light on these processes. In particular, comparative research between *D. melanogaster*, *Ae. aegypti* and *An. gambiae* may provide insight into receptor expression patterns on the olfactory appendages. Sensilla and associated OSNs show strong stereotypy and topographic organization on the antennae of *D. melanogaster*, and similar processes may be occurring in *An. gambiae* [74]. In addition, in *D. melanogaster*, *Ae. aegypti* and *An. gambiae*, some OSNs can coexpress ORs, IRs or OR–IR receptor genes [75]. It remains to be determined how odorants that activate different types of receptors interact when detected by the same neuron. This co-expression could represent a mechanism of olfactory coincidence detection, allowing a single neuron to integrate multiple olfactory signals simultaneously, a process that may be particularly relevant for discriminating between chemically similar plants or hosts.

Beyond receptors expressed in the same neuron, neurons within the same sensillum may also interact to influence their olfactory responses. These interactions are not chemically mediated but rather through near-field changes in the electrical current between neurons in the sensillum, called ephaptic coupling. This causes an activated OSN to suppress the firing rate of the other OSNs, as shown in capitate peg neurons in *Ae. aegypti* and *An. gambiae* [59,76]. This within-sensillum processing may be critical for contrast enhancement of certain odorants in a mixture.

#### (ii) Antennal lobe representation and processing of plant odours

The ALs are organized into dense regions of neuropil, called glomeruli. In *Ae. aegypti* and *An. gambiae*, there are approximately 63 and 67−70 glomeruli, respectively, with no clear differences in glomerular number between sexes [77,78]. In contrast, in the *Cx. quinquefasciatus* AL, there are an estimated 62 and 44 glomeruli in the female and male, respectively [79]. OSNs expressing the same receptors project their axons to a glomerulus where they synapse with projection neurons (PNs) and local neurons (LNs) [80] (figure 3B). While LNs may arborize across multiple glomeruli without projecting axons outside the ALs, PNs project their axons to higher brain areas, including the mushroom bodies (MBs) and lateral horns (LHs) (figure 3B).

In the ALs, activity maps can be established using calcium imaging, which reflect the responses of OSNs projecting their axons to the glomeruli. Each odour is associated with a unique pattern of glomerular activity, indicating that specific plant volatiles activate specific glomeruli. In *Ae. aegypti*, it has been shown that the temporal dynamics and spatial patterns of glomerular activity play critical roles in encoding key odour features, such as odour identity and concentration [19]. Indeed, in both *Aedes increpitus* and *Ae. aegypti*, a strong, floral-evoked multiglomerular activity pattern was observed in the anterior-medial glomeruli (AM2, AM3), as well as in the anterior-lateral glomeruli (AL3, LC2). For *Ae. aegypti*, the anterior-medial glomeruli are sensitive to terpenes, such as lilac aldehyde, whereas aliphatic aldehydes (nonanal) activate the anterior and lateral-central glomeruli while inhibiting the anterior-medial glomerulus [19] (figure 3C).

Lahondère *et al.* also highlighted the important role of lateral inhibition mediated by the neurotransmitter γ-aminobutyric acid (GABA) in the representation of odours in the AL [19]. An odorant can drive responses in a certain glomerulus and, via the GABAergic system, inhibit others simultaneously. For example, a high concentration of nonanal will increase the activation of the LC2 glomerulus while suppressing the AM2 glomerulus response to lilac aldehyde. Conversely, an increasing concentration of lilac aldehyde will increase the activation of the AM2 glomerulus and induce suppression of the LC2 glomerulus. This excitatory/inhibitory balance enhances the perception of weak but behaviourally relevant odours while attenuating signals coming from background or repellent odours and is crucial for processing complex odours, such as those emitted by flowers (figure 3D,E).

In *Ae. aegypti*, the representation of human odour is shaped by combinatorial coding at the level of the ALs, specifically involving two identified glomeruli, H and B. The B glomerulus responds reliably to common compounds found in the odours of vertebrate hosts (humans and animals alike), whereas the H glomerulus is selectively activated by long-chain aldehydes, present in high proportions in human odour [81]. This activation is prolonged, constituting the most specific neuronal signature of human odour observed in the OR-Orco pathway. It is the combined coactivation of these two glomeruli that produces a robust representation of human odour [81]. The study by Zhao *et al.* [81] also provides valuable insights parallel to those of Lahondère *et al.* [19]. Both studies show that relatively few glomeruli represent complex odours despite the presence of tens to hundreds of compounds in the bouquet. In Lahondère *et al.’*s study, this glomerular sparsity was due to GABAergic inhibition suppressing the activity of other glomeruli and associated olfactory inputs [19].

#### (iii) Projection neurons and local neurons representation and processing of plant odours

The neuroanatomy and cellular organization of the mosquito’s AL are similar to those of *D. melanogaster* but exhibit some differences [82]. Beyond the signal input from the OSNs, odour representation in the AL also reflects a combinatorial and spatio-temporal coding of odours by PNs and LNs, allowing for the precise and efficient encoding of odour identity, including plant-derived odorants such as limonene, geranyl acetate and methyl salicylate in *Ae. aegypti* [82].

Indeed, results have suggested the PN population contributes to a combinatorial representation of odour: although some neurons respond to multiple compounds, it is the collective and dynamic ensemble activation that encodes odour identity. In *Ae. aegypti*, approximately 40–50 PNs are sufficient to discriminate an odour in less than 700 ms [82]. Even among neurons associated with the same glomerulus, response variability is observed, suggesting intra-glomerular functional diversity that could enhance the precision and robustness of neuronal coding of complex floral odours [82]. Some PNs can exhibit excitatory responses, others inhibitory responses, while others display biphasic responses, with an excitatory phase followed by an inhibitory phase. The temporal dynamics of the PNs may reflect LN inhibition [83] or intrinsic properties of the PNs.

In parallel, LNs play a fundamental role in modulating glomerular activity and show robust responses to odours. In *Ae. aegypti*, LNs show high variability in selectivity, with heterogeneous response profiles. Some LNs displayed repeatable subthreshold changes in membrane potential when stimulated with different odorants, suggesting they receive diverse olfactory inputs [82]. Pan-glomerular LNs, which innervate the entire AL, exhibit functional homogeneity and may act as global modulators [82], whereas other LN subtypes show select glomerular innervation, perhaps reinforcing or inhibiting specific glomerular responses. Similar to *D. melanogaster*, a handful of LNs are not GABAergic [82], suggesting they may be glutamatergic and inhibitory or cholinergic and excitatory. Paired recordings of PNs revealed the possibility of lateral excitation from one PN to another, which could be mediated by excitatory LNs [82]. These findings may suggest a dual role for LNs in both inhibiting and facilitating selective glomerular excitation.

#### (iv) Higher order brain regions and processing of plant odours

Little is known about the integration and representation of olfactory information in the protocerebrum. In *D. melanogaster*, it is known that these regions play an essential role in both learnt (i.e. MB) and innate (i.e. LH) olfactory behaviours [84,85]. In *Ae. aegypti*, PNs innervating dorsomedial glomeruli of the antennal lobe project mainly to the dorsal region of the LH, and those innervating other glomeruli project to ventral and anterior regions [82], perhaps causing a functional map within the LH that is related to odour valence. However, this odour categorization remains to be demonstrated. In the future, a connectome and cell-specific lines will enable increased understanding of how mosquitoes translate olfactory information into behavioural decisions.

## 4. Control interventions using plant odours

**Figure 4.**
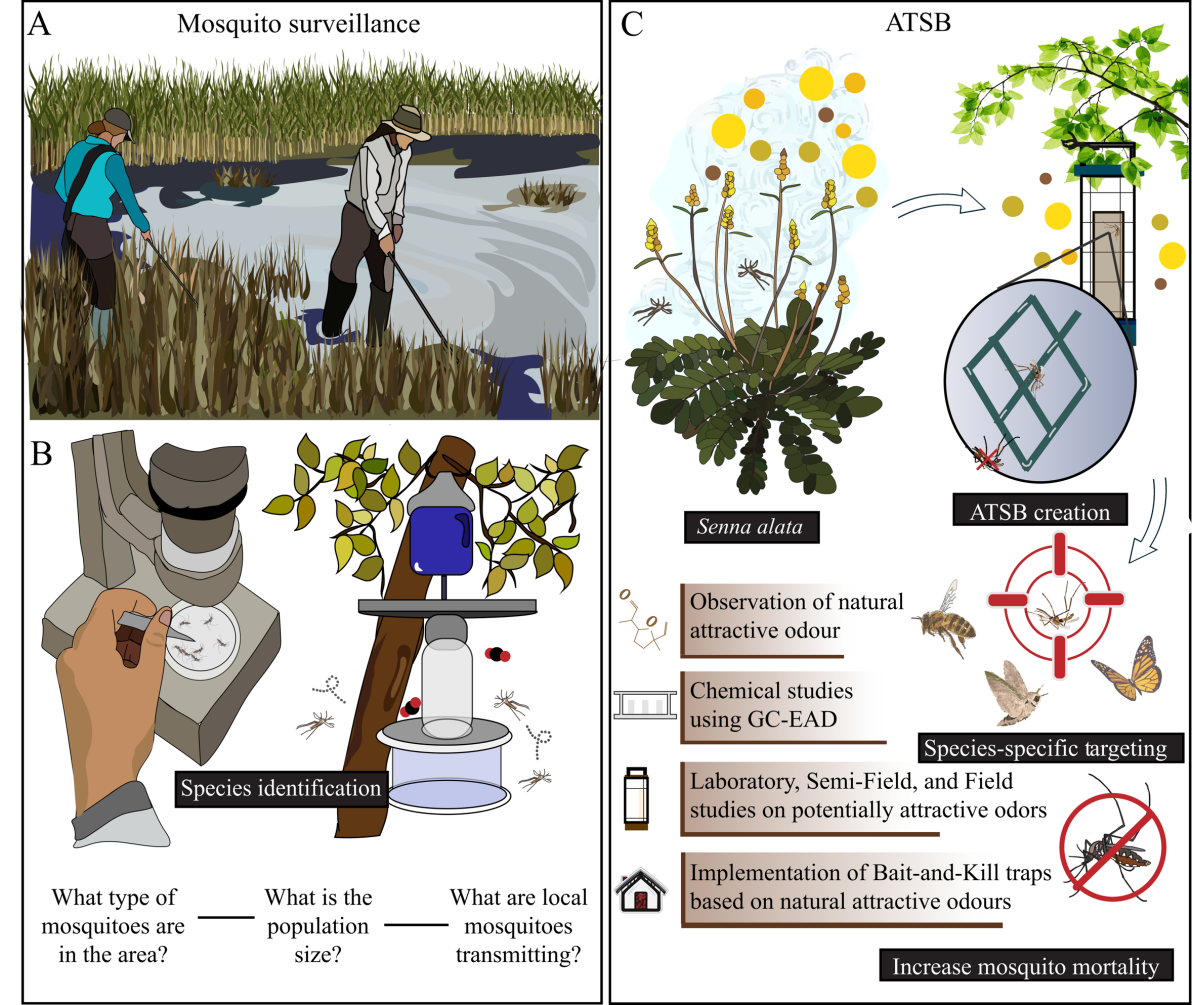
Integration of plant-derived volatiles into mosquito surveillance and control. Plant-derived attractants can be integrated into public health efforts, including the development of ATSB systems, the identification and classification of mosquito species, and the monitoring of mosquitoes in natural habitats. (A) Field researchers conducting mosquito surveillance in natural environments. (B) Mosquito traps and associated attractants derived from plant odours can be used for the monitoring of mosquito species. (C) Integration of plant odours into attractant lures for ATSB systems. Images generated using Adobe Illustrator.

Over the last decade, effective mosquito control strategies have incorporated multiple techniques collectively known as integrated pest management strategies (IPM) (figure 4). These strategies typically include mosquito surveillance, physical control and habitat reduction, larval and adult mosquito control, insecticide resistance monitoring, public education and data collection [86]. Of these control strategies, adult mosquito control techniques have been improved by a deeper understanding of mosquitoes’ attraction to plant odours, allowing the targeting of both male and female mosquitoes during their feeding on plant nutrients. Investigating the underlying mechanisms that drive mosquito behaviour in response to plant-emitted compounds could enhance long-term vector control strategies [87] (figure 4).

### (a) Semi-field and field studies in prioritized species

ATSBs exploit the innate foraging behaviour of mosquitoes to control local vector populations [88] (figure 4E). The combination of an attractive substance, usually in the form of a natural product such as fruit juice, with a toxin-laced sugar solution has been tested in diverse field settings against some of the most important vector species. Over the past two decades, ATSB integration into vector management strategies has suggested efficacy in reducing populations of some of the most important vector genera [89]. ATSBs also have significant implications for mitigating the negative impact of traditional vector control methods, such as insecticides. The widespread application of insecticides has led to the development of multiple mechanisms of insecticide resistance, eroding their effectiveness. Efforts are urgently needed to develop novel methods of vector control that facilitate reductions in area-wide insecticide applications. The use of ATSBs is an alternative to traditional methods that has the potential to address many of these concerns [90].

ATSBs have been deployed in bait stations or by spraying vegetation with a formulation of an attractant lure and toxic insecticide. ATSBs deployed by spraying non-flowering vegetation have been shown to reduce *Ae. aegypti* and *Ae. albopictus* populations. For example, treatment sites in residential areas of Key Largo, Florida, had up to 80% reductions in *Ae. aegypti* adult populations compared with pretreatment levels [91], while a spray application in St. Augustine, Florida, resulted in significant reductions in *Ae. albopictus* adults in treated versus control sites [92]. In a study conducted in an arid climate in Israel, ATSB was applied as a juice formulation in sugar-rich and sugar-poor sites. Populations of *Anopheles sergentii* were reduced by over 95% in both locations, demonstrating the effectiveness of ATSB even in sugar-rich environments [89].

The potential efficacy of ATSBs in controlling both male and female mosquitoes has led to field trials using ATSB bait stations outside dwellings. However, in contrast to the studies cited above, the results have shown variable outcomes. In Mali, the combined deployment of ATSBs and long-lasting insecticidal nets was conducted among 14 villages, resulting in a significant reduction in *An. gambiae s.l.* densities by as much as 70% compared with control sites, and reduced the calculated entomological inoculation rates [93]. However, more recent Phase III trials in Zambia, Kenya and Mali, which collectively involved approximately 5000 person-hours and enabled testing across seasons and between mosquito species (*Anopheles funestus, An. gambiae* and *Anopheles coluzzii*), showed no efficacy of ATSBs [94]. Across all Phase III trials, the clinical malaria incidence, malaria prevalence and mosquito abundance did not differ significantly from those of the control groups. The difference in efficacy between the smaller experiments and the Phase III trials suggests that additional research and approaches, perhaps at the level of the attractant lure and/or experimental design, are needed.

### (b) Limitations of attractive toxic sugar baits

Although the majority of attractants used in ATSBs are fruit-based, few studies have been conducted using bioactive compounds (i.e. identified from GC-EAD) that modulate mosquito behaviour. The imbalance between widespread ATSB usage and limited research on attractant efficacy can ultimately hinder the development of effective mosquito control strategies. A significant weakness in the use of fruit-based attractants is the variation in odour emissions, which can arise from the use of different cultivars, fruit parts, storage conditions and fermentation status [32].

Current formulations often rely on fruit-based syrups or pulp mixtures to evaluate the attractiveness of fruit-derived ATSB lures [89]. However, these formulations can differ widely in their odour emission rates and profiles. For instance, commercially purchased fruit syrups may contain additives that alter odorant emissions, making them unrepresentative of the natural odorants of fruit. Furthermore, some studies ferment fruit attractants, promoting the release of microbe-derived volatiles [95]. This additional technical feature may complicate the interpretation of results, as it becomes difficult to distinguish whether attractive compounds originate from the fruit or microbe.

The variability of using fruit as an attractant necessitates the development of synthetic attractants, which may offer promising alternatives. Synthetic mixtures can mimic nutrient-derived volatiles while controlling the natural variability of fruits. However, the successful development of synthetic mixtures requires behavioural, chemical and electrophysiological validation to determine which compounds are most influential. Currently, only a handful of studies have directly tested the effects of individual fruit odour compounds on mosquito attraction. For example, ripe mango fruits (*Mangifera indica*) elicited strong antennal and behavioural responses to terpene compounds in *An. gambiae* using the GC-EAD system. Rice was detected to elicit strong antennal responses using the GC-EAD system in *Anopheles arabiensis,* and a synthetic mixture was created eliciting short-range attraction and oviposition in gravid females [96]. Future research should use interdisciplinary approaches to develop attractant lures that target specific mosquito species. Ultimately, the disconnect between the widespread use of plant syrups and concentrates and the limited understanding of the chemical drivers of mosquito attraction can obstruct the development of more effective and targeted mosquito control strategies.

## 5. Conclusions

There have been remarkable innovations in mosquito sensory neuroscience and chemical ecology. However, the interface between these two fields has been limited, with relatively few electrophysiologically active odorants from known sources and their cognate odorant receptors identified. The olfactory circuits in the brain that give rise to behavioural attraction to odour sources, including those from plant nutrients, also remain vestigial. Despite these gaps, such insight can provide critical information for developing new control interventions, attractants and repellents. For example, due to the convergence of odorant sensory neurons to AL glomeruli (10−100 : 1 OSN : PN), determining the glomerular representations of host- and plant-nutrient odours could be an effective means of identifying new lures [19,81]. Similar approaches using machine learning and AL responses have been applied to develop repellents potentially more effective than N,N-Diethyl-meta-toluamide (DEET) [97]. At the olfactory receptor level, identifying the receptor ligands could provide new repellents or attractants [59]. The increasing resistance to insecticides and the impacts of climate change on mosquito populations motivate the development of new control interventions. ATSBs offers such an approach, but one that necessitates further research at multiple levels, including the identification of signal molecules and their binding to specific receptors, as well as the encoding of neuronal signals that are processed in the brain to elicit behavioural responses.

## Data Availability

Supplementary material is available online [98].
